# Modelling semi‐attributable toxicity in dual‐agent phase I trials with non‐concurrent drug administration

**DOI:** 10.1002/sim.6912

**Published:** 2016-02-19

**Authors:** Graham M. Wheeler, Michael J. Sweeting, Adrian P. Mander, Shing M. Lee, Ying Kuen K. Cheung

**Affiliations:** ^1^ MRC Biostatistics Unit Hub for Trials Methodology ResearchCambridge Institute of Public HealthForvie Site, Robinson WayCambridgeCB2 0SRU.K.; ^2^ Cardiovascular Epidemiology Unit, Strangeways Research LaboratoryUniversity of CambridgeCambridgeCB1 8RNU.K.; ^3^ Department of Biostatistics, Mailman School of Public HealthColumbia University722 West 168th StreetNew YorkNY 10032U.S.A.

**Keywords:** phase I trials, adaptive designs, Bayesian methods, drug combinations, dose‐toxicity modelling

## Abstract

In oncology, combinations of drugs are often used to improve treatment efficacy and/or reduce harmful side effects. Dual‐agent phase I clinical trials assess drug safety and aim to discover a maximum tolerated dose combination via dose‐escalation; cohorts of patients are given set doses of both drugs and monitored to see if toxic reactions occur. Dose‐escalation decisions for subsequent cohorts are based on the number and severity of observed toxic reactions, and an escalation rule. In a combination trial, drugs may be administered concurrently or non‐concurrently over a treatment cycle. For two drugs given non‐concurrently with overlapping toxicities, toxicities occurring after administration of the first drug yet before administration of the second may be attributed directly to the first drug, whereas toxicities occurring after both drugs have been given some present ambiguity; toxicities may be attributable to the first drug only, the second drug only or the synergistic combination of both. We call this mixture of attributable and non‐attributable toxicity semi‐attributable toxicity. Most published methods assume drugs are given concurrently, which may not be reflective of trials with non‐concurrent drug administration. We incorporate semi‐attributable toxicity into Bayesian modelling for dual‐agent phase I trials with non‐concurrent drug administration and compare the operating characteristics to an approach where this detail is not considered. Simulations based on a trial for non‐concurrent administration of intravesical Cabazitaxel and Cisplatin in early‐stage bladder cancer patients are presented for several scenarios and show that including semi‐attributable toxicity data reduces the number of patients given overly toxic combinations. © 2016 The Authors. *Statistics in Medicine* Published by John Wiley & Sons Ltd.

## 1 Introduction

In oncology, phase I clinical trials are conducted to evaluate the toxicity profile of a novel agent. The aim is to identify the maximum tolerated dose (MTD), defined to be the largest dose that is expected to cause unacceptable toxicity in a specified proportion of patients 1. The desired proportion is known in practice as the target toxicity level (TTL) and is denoted here as *Γ*. What is considered as unacceptable toxicity will depend on the drug, disease and patient population. In practice, unacceptable toxicity is known as dose‐limiting toxicity (DLT) and is usually restricted to the observation of one or more grade 3 or higher toxic reactions, as defined by the National Cancer Institute's Common Terminology Criteria for Adverse Events 2. For trials of cytotoxic drugs, such a dose is assumed to be the most promising for reducing tumour size, with a constrained potential for inducing DLTs in patients.

Combinations of drugs are often required to effectively treat cancer patients. There may be synergistic benefits in combining two or more cytostatic/cytotoxic agents, such as increasing the potential for reducing the size of tumours 3. In addition, with more advanced molecularly targeted therapies, different drugs that form a treatment regimen may be used to deal with cellular heterogeneity within tumours, thus effectively combating tumours before they become resistant to drugs 4. Combinations of chemotherapeutic drugs may be administered concurrently or non‐concurrently over a treatment cycle, and such a choice is usually dependent on the disease being treated, the treatments being used, and the biological mechanism via which the treatments act 5, 6, 7, 8, 9. Administering treatments concurrently is often undertaken in order to quickly kill tumour cells and/or prevent remaining tumour cells from developing immunity to particular drugs and thus improve treatment efficacy with respect to disease‐free survival and overall survival 10. However, whilst concurrent administration may be clinically efficacious, it may lead to severe toxicities in patients because of the large doses and high dose‐intensities that patients receive. Administering drugs non‐concurrently over a cycle may reduce the likelihood of patients experiencing severe toxic reactions whilst still providing the clinical benefit of combining treatments to treat tumours 3.

In a trial, clinicians are responsible for determining whether any observed toxicity is attributable to one or more of the treatments, or a consequence of disease progression 11. In a combination trial of two known drugs, the issue of misattributing toxicity to an incorrect source (that is, attributing a treatment‐related toxicity to disease, or a disease‐related toxicity to treatment) is less likely, because both drugs have been studied previously and their toxicity profiles are reasonably well known. However, in dual‐agent phase I trials of two drugs with similar toxicity profiles, there is the issue of drug‐related *non‐attributable* (NA) toxicity (for the remainder of this work, toxicity is that caused by the experimental agents, and disease‐related toxicity is ignored). In single‐agent trials where only one drug is escalated and no other therapies are administered, a DLT observed in a patient is due to the dose of that particular drug, that is, the DLT is *attributable* to the drug. In dual‐agent trials however, there possibly exists further ambiguity. Because two drugs are being administered, depending on the trial context, it is possible that one cannot attribute a DLT to a particular agent under investigation. One may say that such a DLT is NA. The situation may be even more complex than this; there is the possibility of a synergistic interaction between both drugs that leads to toxicity even though each drug given alone is deemed safe 12. Yin and Yuan 13 considered modelling the four possible toxicity outcomes (DLT/no DLT due to drug *A* coupled with DLT/no DLT due to drug *B*) via a contingency table approach, which can be used when there are non‐overlapping toxicities for drugs *A* and *B*. When there are overlapping toxicities and toxicities that cannot be attributed to specific drugs, the outcomes can be collapsed into a simpler DLT/no DLT outcome for each combination.

Consider an example trial of two drugs *A* and *B*, again with overlapping toxicities, where the aim is to identify the MTD combination with respect to the occurrence of first‐cycle DLTs that are drug related (i.e. we do not consider disease‐related toxicity). In such a trial, drug *A* is administered at the start of the treatment cycle, and drug *B* is administered at a much later time point within the cycle (e.g. several days later) if and only if the patient does not experience a DLT after receiving drug *A*. Any DLT observed before drug *B* is administered is attributable to drug *A* only. However, after a patient receives drug *B*, any observed toxicity may be due to drug *A*(in the form of delayed toxicity), drug *B* or a synergistic combination of both drug *A* and drug *B*, as mentioned previously. Such toxicity may be regarded as NA. This example trial involves attributable and NA toxicity occurring; we define this mixture of attributable and NA toxicity as *semi‐attributable* (SA) toxicity. By incorporating details of when doses are administered and whether a DLT was observed before or after drug *B* was given, if at all, we may be able to better determine whether drug *A* and/or drug *B* should be escalated and ideally avoid early onset toxicities from drug *A* alone, meaning more patients are likely to receive the full dose combination that is believed to be more efficacious than each treatment given as monotherapies.

The inclusion of non‐concurrent drug administration has yet to be considered in statistical–methodological research for phase I trials, and no novel designs for combination trials have incorporated this detail. Therefore, we propose methodology for designing a dual‐agent phase I trial of treatments with overlapping toxicities, where it is not clear whether drug *A*, drug *B* or both drugs are responsible for causing toxicities, and these treatments are given non‐concurrently. Section 2 describes a real‐life dual‐agent phase I trial that motivates this work, and Section 3 presents a method for a trial where the doses of both drugs can be adapted between patients in order to estimate one or more MTD combinations. Section 4 details a simulation study that compares our work with a design that assumes drugs are being given concurrently, and Section 5 describes the results with respect to both accuracy in MTD combination recommendations and chance of dosing patients at unsafe dose combinations. We conclude this paper with a summary of our work, including limitations and areas of further research.

## 2 Motivational trial

The work in this paper is motivated by a submitted protocol for a dual‐agent phase I dose‐escalation trial featuring the non‐concurrent administration of Cabazitaxel (*A*) and Cisplatin (*B*) intravesically (via a catheter into the bladder) in patients diagnosed with recurrent high‐risk non‐muscle invasive bladder cancer (at stages tumour *in situ*, Ta or T1) who have previously received standard treatment of intravesical *Bacillus* Calmette–Guérin (Clinical Trials.gov Identifier NCT02202772). Both drugs have similar toxicities associated with intravenous administration (urinary tract infections, renal problems and nausea), and it is believed that this will also be the case for intravesical administration. Treatment cycles are weekly (7days), with Cabazitaxel being administered to patients on the morning of day 1 and Cisplatin being administered on the morning of day 5 only if no DLT attributable to Cabazitaxel is observed in the patient before the administration of Cisplatin. Therefore, any DLT in the first cycle occurring before Cisplatin is administered is due to Cabazitaxel, whereas a DLT occurring after the administration of Cisplatin may be due to Cabazitaxel alone, Cisplatin alone or a combination of the two. Patients will receive a maximum of 6weeks of treatment. A 2000mg/100ml dose of gemcitabine is also administered to patients during the treatment cycle (on the morning of day 3), which has previously been shown to be well tolerated when given intravesically at this concentration to patients with non‐muscle invasive bladder cancer 14 (we do not consider modelling the fixed dose of gemcitabine in our work, but if we were to, our modelling approach would be amended accordingly; see discussion). Initially, the toxicity profile of a 2 × 4 dose combination grid formed by two dose levels of Cabazitaxel ({*a*_1_,*a*_2_}={2.5,5}mg/100ml) and four dose levels of Cisplatin ({*b*_1_,*b*_2_,*b*_3_,*b*_4_}={0,66,80,100}mg/100ml) was to be investigated. However, this was later amended to be five dose combinations from this set of eight ({(*a*_1_,*b*_1_),(*a*_2_,*b*_1_),(*a*_2_,*b*_2_),(*a*_2_,*b*_3_),(*a*_2_,*b*_4_)}) because of sample size limitations. The definition of DLT is deemed to be the observation of excessive toxicity (at least one grade 3 or grade 4 toxicity as defined per the National Cancer Institute's Common Terminology Criteria for Adverse Events) in the first cycle (week) of treatment. In summary, the investigators wish to identify the dose combination that, when given in the schedule stated, has an estimated probability of DLT over a cycle close to 0.25. Using this as a motivational study, we consider how a dual‐agent dose‐escalation study with non‐concurrent drug administration can be designed so that exploration of a full dose‐toxicity surface, whilst reducing dosing at overly toxic combinations, can be achieved and compare its operating characteristics to an existing approach that does not account for SA toxicity.

## 3 Methods

We present the methodology proposed to incorporate SA toxicity for dual‐agent phase I dose‐escalation trials of non‐concurrently administered drugs, as well as the approach where such detail is omitted (an entirely NA approach).

### 3.1 Semi‐attributable toxicity

**Figure 1:**
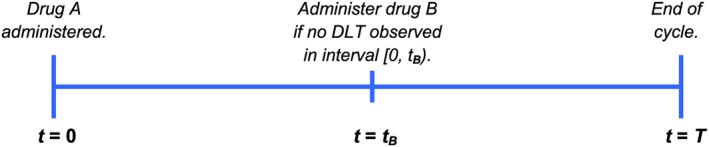
Timeline detailing administration of agents *A* and *B* for a single patient over time interval [0,*T*]. DLT, dose‐limiting toxicity.

Consider a dual‐agent trial where *a*_*j*_ denotes the *j* ^*th*^ dose level of drug *A* {j=1,…,J} and *b*_*k*_ denotes the *k* ^*th*^ dose level of drug *B* {k=1,…,K}. For each patient, drug *A* is administered at time 0, and drug *B* is administered at pre‐planned time *t*_*B*_ provided no DLT has been observed in the patient before time *t*_*B*_. An entire cycle is observed for the time window [0,*T*], with *t*_*B*_<*T*(Figure 1). To incorporate the concept of SA toxicity as defined in Section 1, let *Y*_*i*_ be a trinary outcome variable for patient *i* such that

(1) $$Y_{i}=\left\{\begin{array}{cc} \;0 & \text{if patient}i\text{does not have a DLT in time interval}\;\left[0,T\right]\\ \;1 & \text{if patient}i\text{has a DLT in time interval}\;\left[0,t_{B}\right)\\ \;2 & \text{if patient}i\text{has a DLT in time interval}\;\left[t_{B},T\right]. \end{array}\right.$$

Let $\pi_{t_{B}}$ be a function such that $\pi_{t_{B}}(a_{j};t_{B},\psi )$ denotes $P\left(Y_{i}=1\;|\;a_{j};t_{B},\psi \right)$, the probability of observing a DLT before time *t*_*B*_ when drug *A* is given alone conditional on dose level *a*_*j*_ and vector of model parameters ***ψ***. The following conditions must hold for $\pi_{t_{B}}$:

- $\pi_{t_{B}}(0;t_{B},\psi )=0$ and
- $\pi_{t_{B}}(a_{j+1};t_{B},\psi )\geq \pi_{t_{B}}\left(a_{j};t_{B},\psi \right)$.

Condition (ii) states the assumption of monotonicity used in dose‐escalation studies, that is, holding *t*_*B*_ and parameters ***ψ*** fixed, the probability of DLT is non‐decreasing as the dose of drug *A* increases. Let *π*_*T*_(*a*_*j*_,*b*_*k*_;***θ***) be the probability of DLT at combination (*a*_*j*_,*b*_*k*_) over time interval [0,*T*], that is, $P(Y_{i}=1\;|\;a_{j};t_{B},\theta )+P(Y_{i}=2\;|\;a_{j},b_{k};t_{B},\theta )$. Here, ***θ*** denotes the vector of model parameters for *π*_*T*_, with *π*_*T*_ satisfying the following conditions:

*π*_*T*_(0,0;***θ***) = 0,
$\pi_{T}(a_{j},b_{k};\theta )\geq \pi_{t_{B}}\left(a_{j};t_{B},\psi \right)$, for all $b_{k}\geq 0$ and $0\leq t_{B}\leq T$,
$\pi_{T}(a_{j+1},b_{k};\theta )\geq \pi_{T}\left(a_{j},b_{k};\theta \right)$ and
$\pi_{T}(a_{j},b_{k+1};\theta )\geq \pi_{T}\left(a_{j},b_{k};\theta \right)$.

Condition (iv) states that the addition of any dose of another agent (i.e. drug *B*), whilst the dose of drug *A* is held constant will give a combination with probability of DLT over time window [0,*T*] greater or equal to that of drug *A* given alone when observed over time window [0,*t*_*B*_). Conditions (v) and (vi) are the assumption of monotonicity (condition (ii)) for drug *A* and drug *B*, respectively. Let ${\bar{\pi }}_{T}=1-\pi_{T}$. Therefore, ${\bar{\pi }}_{T}\left(a_{j},b_{k};\theta \right)=P\left(Y_{i}=0\;|\;a_{j},b_{k};\theta \right)$, the probability of not observing a DLT in the time period [0,*T*] at dose combination (*a*_*j*_,*b*_*k*_). Within this context, one may assume that $\pi_{t_{B}}$ and *π*_*T*_ are related in some way. One simplifying assumption is that $\pi_{t_{B}}$ is linearly related to *π*_*T*_, that is, $\pi_{t_{B}}=\lambda \pi_{T}$. Under this assumption, ***ψ***={***θ***,*λ*}. Therefore,

(2) $$\pi_{t_{B}}\left(a_{j};t_{B},\psi \right)=\pi_{t_{B}}\left(a_{j};t_{B},\theta ,\lambda \right)=\lambda \pi_{T}\left(a_{j},0;\theta \right),$$

where 0≤λ<1 is a fraction that can be estimated in the model, or fixed at $\frac{t_{B}}{T}$, say. Although it is unrealistic that *λ* will ever equal 1, this scenario may be understood as observing no DLTs after time *t*_*B*_ due to drug *A*, with drug *B* never being administered in the first cycle. If we were permitted to vary *t*_*B*_ in our trial, then such a situation would be easier to interpret. However, we assume for this trial that *t*_*B*_ is fixed.

The previous assumption relating $\pi_{t_{B}}$ to *π*_*T*_ means that only a choice of probability function for *π*_*T*_ is required, and thus, *π*_*T*_ shares the same parameter vector ***θ*** with $\pi_{t_{B}}$, but with $\pi_{t_{B}}$ also dependent on *λ*. This is valid because *π*_*T*_(*a*_*j*_,0;***θ***) is the probability of DLT over the interval [0,*T*] solely due to drug *A* and thus must be greater than or equal to $\pi_{t_{B}}(a_{j};\psi )$, which satisfies condition (ii) previously. Under this specification, the probabilities relating to each outcome (*Y*_*i*_=0,1,or2) being observed are modelled as follows:

(3) $$P\left(Y_{i}=0\;|\;a_{j},b_{k};\psi \right)={\bar{\pi }}_{T}\left(a_{j},b_{k};\theta \right),$$

(4) $$P\left(Y_{i}=1\;|\;a_{j};t_{B},\psi \right)=\pi_{t_{B}}\left(a_{j};\psi \right)=\lambda \pi_{T}\left(a_{j},0;\theta \right)\;\text{and}$$

(5) $$P\left(Y_{i}=2\;|\;a_{j},b_{k};t_{B},\psi \right)=\pi_{T}\left(a_{j},b_{k};\theta \right)-\lambda \pi_{T}\left(a_{j},0;\theta \right).$$

It can easily be shown that *π*_*T*_(*a*_*j*_,*b*_*k*_;***θ***) − *λ* *π*_*T*_(*a*_*j*_,0;***θ***) is non‐negative. Because 0≤λ<1, under the assumption of monotonicity, $\pi_{T}\left(a_{j},b_{k};\theta \right)\geq \pi_{T}\left(a_{j},0;\theta \right)>\lambda \pi_{T}\left(a_{j},0;\theta \right)$. Given the probability function choices stated previously, the outcome *Y*_*i*_ for patient *i* has a categorical distribution with the following probabilities:

(6) $$Y_{i}∼\text{Cat}\left(3,{\bar{\pi }}_{T}\left(a(i),b(i);\theta \right),\lambda \pi_{T}\left(a(i),0;\theta \right),\pi_{T}\left(a(i),b(i);\theta \right)-\lambda \pi_{T}\left(a(i),0;\theta \right)\right),$$

where *a*(*i*) denotes the dose of drug *A* given to patient *i* and *b*(*i*) denotes the dose of drug *B* given to patient *i* (if at all). Therefore, the likelihood contribution of patient *i* to the overall likelihood is as follows:

(7) $$\begin{array}{cc} L\left(\psi \;|\;Y_{i},a(i),b(i)\right)= & \;{\bar{\pi }}_{T}{\left(a(i),b(i);\theta \right)}^{\left[Y_{i}=0\right]}\pi_{t_{B}}{\left(a(i);\psi \right)}^{\left[Y_{i}=1\right]}{\left\{{\bar{\pi }}_{t_{B}}\;(a(i);\psi )-{\bar{\pi }}_{T}(a(i),b(i);\theta )\right\}}^{\left[Y_{i}=2\right]}\\ = & {\left\{1-\pi_{T}(a(i),b(i);\theta )\right\}}^{\left[Y_{i}=0\right]}{\left\{\lambda \pi_{T}(a(i),0;\theta )\right\}}^{\left[Y_{i}=1\right]}\\ \times {\left\{\pi_{T}(a(i),b(i);\theta )-\lambda \pi_{T}(a(i),0;\theta )\right\}}^{\left[Y_{i}=2\right]}, \end{array}$$

where [*Y*_*i*_=*y*] is the Iverson bracket, which takes value 1 if *Y*_*i*_=*y*, where *y*∈{0,1,2}, and 0 otherwise. Therefore, after observing *n* patients, the overall likelihood is $L(\psi \;|\;D_{n})=\overset{n}{\underset{i=1}{\prod }}L(\psi \;|\;Y_{i},a(i),b(i))$, where $D_{n}$ denotes the set of all accrued trial data, that is, dose combinations (*a*(*i*),*b*(*i*)) and binary DLT responses *Y*_*i*_, given to patients i={1,…,n}. Using Bayes' theorem, with prior distribution *f*(***ψ***) for parameter vector ***ψ***, the posterior distribution of ***ψ***, denoted $g\left(\psi \;|\;D_{n}\right)$, is as follows:

(8) $$g(\psi \;|\;D_{n})=\frac{f(\psi )L(\psi \;|\;D_{n})}{\underset{\Psi }{\int }f(\psi )L(\psi \;|\;D_{n})\;d\psi }.$$

Once $g\left(\psi \;|\;D_{n}\right)$ has been calculated, the posterior distributions for parameters contained in ***ψ*** can be used to compute the posterior distribution of *π*_*T*_(*a*_*j*_,*b*_*k*_;***θ***), the probability of DLT at dose combination (*a*_*j*_,*b*_*k*_) over the interval [0,*T*], for all dose combinations. Because the investigators wish to identify the combination (*a*(∗),*b*(∗)) such that

(9) $$(a(*),b(*))=\arg\underset{\;\left(a_{j},b_{k}\right)}{\min}\left|\pi_{T}\left(a_{j},b_{k};\theta \right)-\Gamma \right|,$$

the aforementioned metric can be used to determine the next dose‐escalation step from patient *n* to patient *n* + 1. Specifically, let N(a(n),b(n)) be the *neighbourhood* of dose combination (*a*(*n*),*b*(*n*)), that is, all dose combinations in the dose combination grid immediately adjacent (vertically, horizontally and diagonally) to the combination given to patient *n*. Then the dose combination for patient *n* + 1 may be expressed mathematically as follows:

(10) $$\left(a(n+1),b(n+1)\right)=\arg\underset{\;\left(a_{j},b_{k}\right)\in N\left(a(n),b(n)\right)}{\min}\left|{\hat{\pi }}_{T}\left(a_{j},b_{k};\theta ,D_{n}\right)-\Gamma \right|,$$

where ${\hat{\pi }}_{T}(a_{j},b_{k};\theta )$ could be chosen to be, say, the posterior median of *π*_*T*_(*a*_*j*_,*b*_*k*_;***θ***). In the case where two dose combinations are equally close to the TTL on the probability scale, one may consider choosing the combination with the smallest dose *a*_*j*_ of drug *A*, because this minimizes *π*_*T*_(*a*_*j*_;***ψ***) and we ideally want patients to receive both drugs in such a trial, although other approaches proposed by investigators may be considered. The constraint of dosing in the neighbourhood of the previously administered dose combination can also be dropped if investigators are happy to make larger changes to doses of each agent than one‐level increase/decreases.

### 3.2 Comparison to non‐attributable toxicity

The previous model construction describes the idea of SA toxicity when drugs are administered non‐concurrently. This may be compared with the simpler setting where differentiation between whether a DLT observed in patient *i* occurred before *t*_*B*_ or afterwards is not considered. Under such a scenario, only the fact of whether a DLT occurred or not within the interval [0,*T*], that is, whether *Y*_*i*_≠0 or whether *Y*_*i*_=0, would be utilized. Furthermore, even if a DLT occurs in patient *i* before *t*_*B*_ and thus drug *B* is not administered, *b*(*i*) is recorded as the dose that would have been given. This is simply referred to as the NA approach. Therefore, the NA approach only considers whether a DLT occurred or not at a combination and does not incorporate any information regarding which agent(s) caused toxicity, or whether drug *B* was given or not. The likelihood under the NA approach for patient *i* is as follows:

(11) $$L(\theta \;|\;Y_{i},a(i),b(i))={\left\{1-\pi_{T}(a(i),b(i);\theta )\right\}}^{\left[Y_{i}=0\right]}{\left\{\pi_{T}(a(i),b(i);\theta )\right\}}^{\left[Y_{i}\neq 0\right]},$$

where *a*(*i*) and *b*(*i*) are the doses of drugs *A* and *B* that patient *i* is due to receive and the prior distribution *h*(***θ***) on ***θ*** is used to obtain posterior distribution $g\left(\theta \;|\;D_{n}\right)$ for parameter vector ***θ***. The likelihood contributions under the SA and NA approaches for patient *i* given DLT outcome *Y*_*i*_=*y* for *y*={0,1,2} are given in Table 1.

**Table 1:** Likelihood contribution of patient *i* dependent on modelling of toxicity and dose‐limiting toxicity outcome.

Method	Response of patient *i*		
	*Y*_*i*_=0	*Y*_*i*_=1	*Y*_*i*_=2
Semi‐attributable	1 − *π*_*T*_(*a*(*i*),*b*(*i*);***θ***)	*λ* *π*_*T*_(*a*(*i*),0;***θ***)	*π*_*T*_(*a*(*i*),*b*(*i*);***θ***) − *λ* *π*_*T*_(*a*(*i*),0;***θ***)
	*Y*_*i*_=0	*Y*_*i*_≠0	
Non‐attributable	1 − *π*_*T*_(*a*(*i*),*b*(*i*);***θ***)	*π*_*T*_(*a*(*i*),*b*(*i*);***θ***)	

In the case where multiple dose combinations are equally close to the TTL on the probability scale (call this set C), one may use weighted randomization to choose a dose combination 15, where each dose combination selection probability is weighted by $n_{c}^{-1}$, the inverse of the number of patients treated at each candidate combination c∈C, that is,

(12) $$P\left(\text{Next cohort given}\left(a_{j},b_{k}\right)|\left(a_{j},b_{k}\right)\in C\right)=\frac{n_{\mathrm{jk}}^{-1}}{\underset{c\in C}{\sum }n_{c}^{-1}}.$$

### 3.3 Dose‐escalation algorithm

Here, we present a summary of the dose‐escalation algorithm to be used in the simulation study. Assume there are a maximum of *N* patients available, who will be enrolled in cohorts of size *c* such that *N* is divisible by *c*. Based on the previous discussion and methodology outlined, dose escalation/de‐escalation proceeds as follows:

1. For *n* = 0, dose the first cohort of *c* patients at *a*_1_.
  - For each patient *i*∈{1,…,*c*}, if patient *i* does not experience a DLT by time *t*_*B*_, dose patient *i* at *b*(*i*) = *b*_1_. Otherwise, do not administer drug *B*. Observe *Y*_1_,…,*Y*_*c*_ and obtain $D_{c}$.
  - Given prior distribution *f*(***ψ***) for ***ψ***, calculate $g\left(\psi \;|\;D_{c}\right)$ and thus posterior distribution of *π*_*T*_(*a*_1_,*b*_1_;***θ***).
  - If $P\left(\pi_{T}\left(a_{1},b_{1};\theta \right)>\Gamma \right)>\tau$, where *τ* is some upper threshold, stop the trial. Otherwise, set *n* = *c*.
2. For n≤N,
  - Let N(a(n),b(n)) be the set of all neighbouring dose combinations to combination (*a*(*n*),*b*(*n*)), the combination prescribed to the previous cohort (including the *n* ^*th*^ person). For all $\left(a_{j},b_{k})\in N(a(n),b(n)\right)$, calculate the posterior probability distribution of *π*_*T*_(*a*_*j*_,*b*_*k*_;***θ***).
  - Identify the dose combination (a(∗),b(∗))∈N(a(n),b(n)) such that for target probability of toxicity *Γ*,
    (13) $$(a(*),b(*))=\arg\underset{\;\left(a_{j},b_{k}\right)\in N(a(n),b(n))}{\min}\left|{\hat{\pi }}_{T}\left(a_{j},b_{k};\theta \right)-\Gamma \right|,$$
    where ${\hat{\pi }}_{T}(a_{j},b_{k};\theta )$ is the posterior median of the distribution *π*_*T*_(*a*_*j*_,*b*_*k*_;***θ***). If there exists more than one such combination that minimizes the aforementioned function, choose the combination that also minimizes *π*_*T*_(*a*_*j*_;***ψ***)(for SA) or use weighted randomization (for NA, see Subsection 3.2).
  - Administer dose level *a*(∗) of drug *A* to patients *i*∈{*n* + 1,…,*n* + *c*}. If patient *i* does not experience a DLT by time *t*_*B*_, administer dose level *b*(∗) of drug *B* to patient *i*. Otherwise, do not administer drug *B*. Observe *Y*_*n* + 1_,…,*Y*_*n* + *c*_ and update $D_{n}$ to $D_{n+c}$.
  - Obtain the posterior distribution $g\left(\psi \;|\;D_{n+c}\right)$. Set *n* = *n* + *c*.
  - If $P\left(\pi_{T}\left(a_{1},b_{1};\theta \right)>\Gamma \right)>\tau$, stop the trial. Otherwise, proceed.
3. If *n* = *N*, the recommended dose combinations at the end of the trial, ℳ, are those that have an estimated posterior median probability of DLT (using the posterior distributions obtained in step (2d)) over the interval [0,*T*] within [*Γ* − *ε*,*Γ* + *ε*], for some small *ε*, and have previously been experimented on during the trial, that is,
  (14) $$ℳ=\left\{\left(a_{j},b_{k}\right)\in D_{N}\;:\;\left|{\hat{\pi }}_{T}(a_{j},b_{k};\theta )-\Gamma \right|\leq \varepsilon \right\}.$$
  Trials that are terminated early for safety concerns or have dose combinations that are suitably close to the TTL but have not been experimented at will not recommend an MTD.

## 4 Simulation study

A simulation study to evaluate the performance of modelling SA toxicity versus NA toxicity in a trial with non‐concurrent administration of Cabazitaxel (*A*) and Cisplatin (*B*), as discussed in Section 2, was conducted, with a TTL of 0.25. All methods were compared on the basis of the percentage of patients that received dose combinations with true DLT probabilities within the interval [*Γ* − *ε*,*Γ* + *ε*], the percentage of patients that received dose combinations with true DLT probabilities much higher than the TTL (commonly known as overdoses) and the distribution of MTD recommendations at the end of the trial. We also consider the mean bias and root mean‐squared error (RMSE) for each model parameter *ν*∈***θ*** and define each of these measures as follows:

(15) $$\text{Mean Bias}=\frac{1}{\left|A\right|}\underset{l\in A}{\sum }\left({\hat{\nu }}_{l}-\nu \right)\;\;\;\;\text{and}\;\;\;\;\;\text{RMSE}=\sqrt{\frac{1}{\left|A\right|}\underset{l\in A}{\sum }{\left({\hat{\nu }}_{l}-\nu \right)}^{2}},$$

where ${\hat{\nu }}_{l}$ is the the posterior median estimate of parameter *ν* at the end of the *l* ^*th*^ trial, A is the set of all trials that did not stop early and |A| is the size of A. We limit these calculations to the set A because trials that do stop early do not yield any MTD combination estimates and parameter estimates are biased towards larger values; the worth of mean bias and RMSE metrics lies in how close parameter estimates (and thus the estimated MTD contour) are to the truth for each scenario.

### 4.1 Dose‐toxicity model

To model the probability of DLT *π*_*T*_(*a*_*j*_,*b*_*k*_;***θ***), we used the Farlie–Gumbel–Morgenstern copula model 16, which has been previously investigated by Yin and Yuan 13, where

(16) $$\pi_{T}\left(a_{j},b_{k};\theta \right)=1-\left(1-p_{j}^{\alpha }\right)\left(1-q_{k}^{\beta }\right)+p_{j}^{\alpha }\left(1-p_{j}^{\alpha }\right)q_{k}^{\beta }\left(1-q_{k}^{\beta }\right)\frac{\exp(\gamma )-1}{\exp(\gamma )+1},$$

which yields

(17) $$\pi_{t_{B}}\left(a_{j};t_{B},\psi \right)=\lambda \pi_{T}\left(a_{j},0;\theta \right)=\lambda p_{j}^{\alpha },$$

where *p*_*j*_ and *q*_*k*_ are skeleton probabilities of DLT for actual dose levels *a*_*j*_ and *b*_*k*_, respectively, when administered as monotherapies, *α* and *β* are non‐negative marginal parameters, γ∈R is an interaction parameter and 0≤λ<1. Therefore, ***ψ***={*α*,*β*,*γ*,*λ*} and ***θ***={*α*,*β*,*γ*}. This model was chosen for its ability to model antagonistic interaction (when *γ* < 0), synergistic interaction (when *γ* > 0) and independent action/no interaction (when *γ* = 0), and also for its parsimony, although other models may be considered 12, 17.

For modelling of SA toxicity, *λ* may be treated as an additional parameter in the model. A sensible choice of prior distribution for *λ* is as follows:

(18) $$\lambda ∼\left\{\begin{array}{ll} \;\text{Beta}\left(\frac{t_{B}}{T-t_{B}},1\right) & \text{if}t_{B}\geq T-t_{B}\\ \;\text{Beta}\left(1,\frac{T-t_{B}}{t_{B}}\right) & \text{if}t_{B}<T-t_{B} \end{array}\right..$$

This choice is recommended because the median of *λ* will be close to $\frac{t_{B}}{T}$, the mean of *λ*, a sensible prior guess of *λ*. Furthermore, such a prior is invariant to the time units that define *T*. Under such a prior, we expect $\lambda >\frac{t_{B}}{T}$ if the toxicities are more likely to occur for drug *A* only, and $\lambda <\frac{t_{B}}{T}$ if there are more toxicities observed after time *t*_*B*_.

### 4.2 Priors

We consider dose‐toxicity scenarios over a 4 × 4 dose combination grid formed by {*a*_1_,*a*_2_,*a*_3_,*a*_4_} and {*b*_1_,*b*_2_,*b*_3_,*b*_4_}, with marginal prior probabilities of DLT {*p*_1_,*p*_2_,*p*_3_,*p*_4_}={0.10,0.15,0.20,0.25} and {*q*_1_,*q*_2_,*q*_3_,*q*_4_}={0.06,0.12,0.18,0.25}. Although larger than the dose combination grid used in the trial discussed in Section 2, this extended grid allows a better assessment of operating characteristics over a wide range of scenarios, particularly those that feature dose combinations with high DLT probabilities. The largest doses of each drug have prior probability of DLT equal to the TTL of 0.25, as it is often the case that the largest dose level of a single drug in a dual‐agent trial is the single‐agent MTD. For parameters of the model in equation (16), uniform priors over the interval [0,2] are proposed for *α* and *β* (similar to those of Yin and Yuan 13) and a normal prior with mean 0 and variance 10 is proposed for *γ*. This is so that *a priori*, the means (and medians) of the marginal parameters are equal to 1, and the mean (and median) of the interaction parameter equal to 0 indicates an assumption of non‐interaction between the two drugs. Furthermore, given the marginal prior probabilities *p*_*j*_ and *q*_*k*_ stated previously, vague prior probability distributions for marginal and joint probabilities of DLT for each drug when given alone and in combination are obtained. In practice, marginal prior distributions may be elicited from clinicians based on monotherapy trials.

A cycle of treatment is considered to be 1week; therefore, for the SA approach, *T* = 7 and *t*_*B*_=4, because we have 4days elapsing between the administration of Cabazitaxel and Cisplatin. Simulations were performed with $\lambda ∼\beta \left(\frac{4}{3},\;1\right)$ so that $E(\lambda )=\frac{t_{B}}{T}=0.571$ and the prior median of *λ* equals 0.595. For NA and SA approaches, the threshold *τ* for determining whether the trial is terminated early is 0.80; as well as being a sensible choice, this threshold also corresponds to terminating the trial early should two DLTs be observed in the first cohort of two patients, regardless of when they occur in the observable interval of [0,*T*]. MTD selection at the end of the trial was determined using the rules outlined in Subsection 3.3 with *ε* = 0.025. This was chosen in order to limit MTD selection to a 5*%* window of probability around the TTL and is also based on other works that implement a similar constraint 15, although the choice of *ε* may be related to the number of dose combinations and also the belief of how flat/steep the dose‐toxicity surface is, based on previous data and expert opinion.

### 4.3 Scenarios

**Figure 2:**
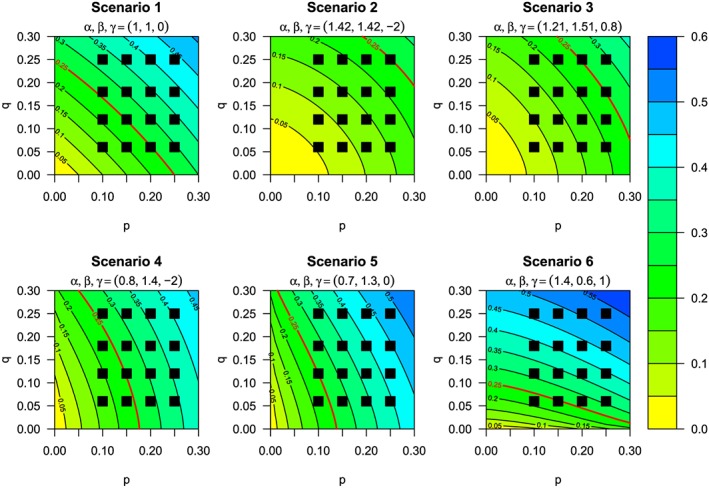
Contour plots of true dose‐toxicity surfaces compared with marginal prior beliefs (*p* and *q*) for scenarios 1–6. Red line indicates maximum tolerated dose contour.

Using the model in Subsection 4.1 and prior distributions specified in Subsection 4.2, we generated six true dose‐toxicity scenarios for our simulation study. The true probabilities of DLT over [0,*T*] per combination under each scenario are specified in Table 2, and the underlying dose‐toxicity surfaces with true parameter values and MTD contour are shown in Figure 2. Scenario 1 is generated by using the prior means/medians of each parameter. Under scenario 2, the MTD is the largest dose combination, with all other combinations deemed safe. Under scenario 3, there are two MTD combinations and one combination above the TTL; furthermore, the dose‐toxicity surface is slightly asymmetric. Scenarios 4, 5 and 6 show very asymmetric dose‐toxicity surfaces: scenario 4 has several combinations on or near the MTD contour, with higher doses of drug *A* more toxic than higher doses of drug *B*; scenario 5 is similar to scenario 4, but with no interaction and one MTD combination at (*a*_1_,*b*_2_); under scenario 6, the increase in toxicity is much faster as drug *B* is escalated relative to when drug *A* is escalated, and half of the 16 dose combinations have a probability of DLT of 0.40 or larger.

**Table 2:** True combination probabilities of DLT over interval [0,*T*] for scenarios 1–6.

Dose level of *B*	Dose level of *A*								
	1	2	3	4		1	2	3	4
	Scenario 1					Scenario 2			
4	0.32	0.36	0.40	0.44		0.17	0.19	0.22	**0.25**
3	**0.26**	0.30	0.34	0.38		0.12	0.15	0.17	0.21
2	0.21	**0.25**	0.30	0.34		0.08	0.11	0.14	0.18
1	0.15	0.20	**0.25**	0.30		0.06	0.08	0.12	0.15
	Scenario 3					Scenario 4			
4	0.18	0.22	**0.25**	0.29		**0.27**	0.32	0.36	0.41
3	0.13	0.17	0.21	**0.25**		**0.23**	0.28	0.33	0.38
2	0.10	0.14	0.18	0.22		0.20	**0.25**	0.31	0.36
1	0.08	0.11	0.16	0.20		0.17	**0.23**	0.29	0.34
	Scenario 5					Scenario 6			
4	0.33	0.39	0.44	0.48		0.46	0.48	0.51	0.53
3	0.29	0.34	0.40	0.45		0.39	0.41	0.43	0.46
2	**0.25**	0.31	0.37	0.42		0.31	0.34	0.36	0.40
1	0.22	0.28	0.34	0.39		0.22	**0.25**	0.28	0.31

MTD combinations shown in bold (those within a 5% window around the target toxicity level (*Γ* = 0.25)).MTD, maximum tolerated dose; DLT, dose‐limiting toxicity.

We also require true probabilities of DLT for $\pi_{t_{B}}(a_{j};\psi )=\lambda \pi_{T}(a_{j},0;\theta )$ in order to conduct our simulation study. All that is required is specification of the true underlying value of *λ*, denoted *λ*_*TR*_. In this simulation study, with *t*_*B*_=4 and *T* = 7, the previous scenarios are investigated with *λ*_*TR*_ equal to either $\frac{2}{14}$, $\frac{8}{14}$ or $\frac{13}{14}$. Under the SA approach where the prior mean of *λ* is $\frac{8}{14}$, setting $\lambda_{\mathrm{TR}}=\frac{2}{14}$ represents a scenario with a lower‐than‐expected probability of DLT in the time interval [0,*t*_*B*_]. Similarly, setting $\lambda_{\mathrm{TR}}=\frac{8}{14}$ represents a scenario with an as‐expected probability of DLT in the time interval [0,*t*_*B*_], and setting $\lambda_{\mathrm{TR}}=\frac{13}{14}$ represents a scenario with a higher‐than‐expected probability of DLT in the time interval [0,*t*_*B*_]. Table 3 displays the different true scenarios for $\pi_{t_{B}}(a_{j};t_{B},\psi )$ under each of the scenarios for *π*_*T*_ given in Table 2, and the varying values of *λ*_*TR*_.

**Table 3:** True marginal probabilities of dose‐limiting toxicity over interval [0,*t*_*B*_] for scenarios 1–6.

Dose level of *A*	Value of *λ*_*TR*_						
	$\frac{2}{14}$	$\frac{8}{14}$	$\frac{13}{14}$		$\frac{2}{14}$	$\frac{8}{14}$	$\frac{13}{14}$
	Scenario 1				Scenario 2		
4	0.04	0.14	0.23		0.02	0.08	0.13
3	0.03	0.11	0.19		0.01	0.06	0.09
2	0.02	0.09	0.14		0.01	0.04	0.06
1	0.01	0.06	0.09		0.01	0.02	0.04
	Scenario 3				Scenario 4		
4	0.03	0.11	0.17		0.05	0.19	0.31
3	0.02	0.08	0.13		0.04	0.16	0.26
2	0.01	0.06	0.09		0.03	0.13	0.20
1	0.01	0.04	0.06		0.02	0.09	0.15
	Scenario 5				Scenario 6		
4	0.05	0.22	0.35		0.02	0.08	0.13
3	0.05	0.19	0.30		0.02	0.06	0.10
2	0.04	0.15	0.25		0.01	0.04	0.07
1	0.03	0.11	0.19		0.01	0.02	0.04

### 4.4 Computational specifications

For each scenario considered (Subsection 4.3), 1000 simulations were run for a maximum of 60 patients, who were dosed in cohorts of two patients. Simulations were conducted in the software package R 18 and OpenBUGS v3.2.2 19 via the BRugs package 20. We use a Gibbs sampling MCMC approach to estimate the posterior distributions of all relevant model parameters, which are used to determine the posterior distributions of the probability that each response is observed at every dose combination. For all simulations, two chains were run, each with a burn‐in period of 500 iterations and posterior sample of 4000 iterations, with thinning occurring every two iterations. Gelman–Rubin plots and autocorrelation plots from OpenBUGS were checked to ensure both chains converged and that autocorrelation was not present.

## 5 Results

We first consider early trial behaviour under both NA and SA approaches and observe how the contour plots of *π*_*T*_(*a*_*j*_,*b*_*k*_;***θ***) change when we observe the response of the first cohort of two patients.

### 5.1 Early trial behaviour

**Figure 3:**
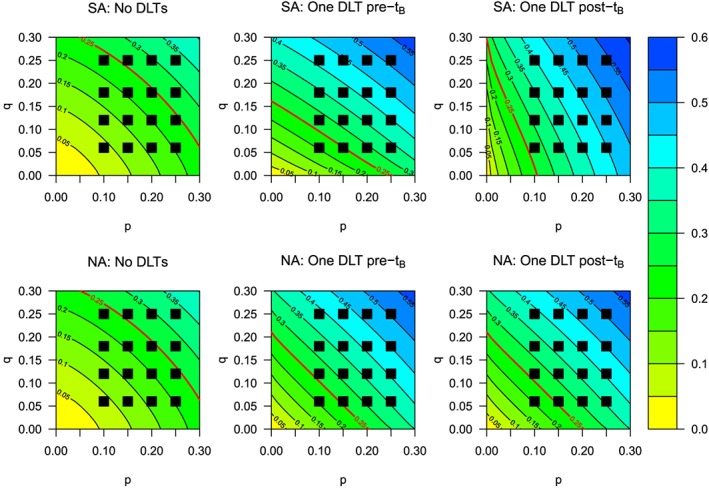
Contour plots for dose‐toxicity surfaces after observing particular dose‐limiting toxicity (DLT) responses for the first two patients under the semi‐attributable (SA) and non‐attributable (NA) approaches, including the estimated maximum tolerated dose contour (red line).

Table 4 shows the dose‐escalation recommendation for patients 3 and 4 given different DLT responses for patients 1 and 2, who receive starting dose (*a*_1_,*b*_1_), under both the NA and SA approaches. We see that the recommendations do not differ between approaches for the first two cohorts but the resultant posterior median parameter estimates that describe the shape of the dose toxicity surfaces are very different (Figure 3). Under the SA approach, observing DLTs before *t*_*B*_ leads to a dose‐toxicity surface that shows drug *A* to be exceedingly toxic; additionally, observing DLTs after time *t*_*B*_, the dose‐toxicity surface reflects a belief that drug *B* is likely to be more toxic than under the NA approach, because no DLT was observed in time interval [0,*t*_*B*_), when the patient had received drug *A* only. This is also shown when comparing the NA approach when (*Y*_1_,*Y*_2_) = (0,1) and the SA approach when (*Y*_1_,*Y*_2_) = (0,2), when a DLT is observed after time *t*_*B*_ in one patient.

**Table 4:** Dose escalation recommendations for patients 3 and 4 after observing different DLT outcomes for patients 1 and 2 under both NA and SA approaches, with respective posterior median parameter estimates.

	NA				SA		
	(*Y*_1_,*Y*_2_)	Dose	(*α*,*β*,*γ*)		(*Y*_1_,*Y*_2_)	Dose	(*α*,*β*,*γ*)
No	(0,0)	(*a*_2_,*b*_2_)	(1.29,1.25,−0.09)		(0,0)	(*a*_2_,*b*_2_)	(1.29,1.12,−0.03)
DLTs							
One	(0,1)	(*a*_1_,*b*_1_)	(0.78,0.80,0.03)		(0,1)	(*a*_1_,*b*_1_)	(0.53,1.16,−0.09)
DLT					(0,2)	(*a*_1_,*b*_1_)	(0.98,0.62,−0.01)
Two	(1,1)	STOP	(0.37,0.42,0.14)		(1,1)	STOP	(0.15,1.00,−0.01)
DLTs					(1,2)	STOP	(0.25,0.63,0.16)
					(2,2)	STOP	(0.82,0.21,0.14)

NA, non‐attributable; SA, semi‐attributable; DLT, dose‐limiting toxicity.

### 5.2 Experimentation and recommendation

**Figure 4:**
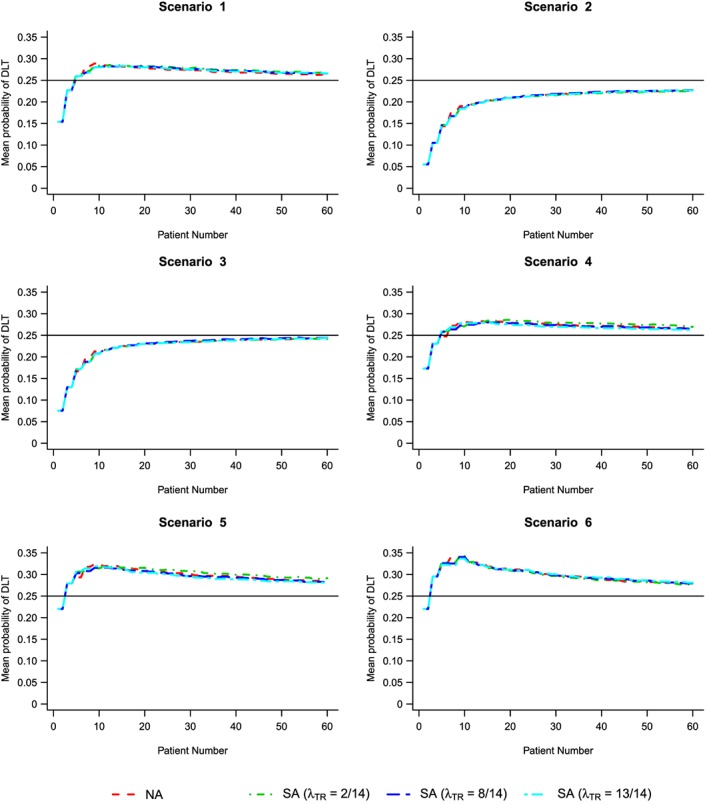
Mean probability of dose‐limiting toxicity (DLT) for each method for scenarios 1–6. Solid horizontal black line indicates target toxicity level *Γ* = 0.25. NA, non‐attributable; SA, semi‐attributable.

Table 5 shows the distribution of patients dosed at combinations with true DLT probabilities falling in certain intervals, as well as the mean and standard deviation of the DLT rates, across all simulations for each dose‐toxicity scenario and value of *λ*_*TR*_, using the NA and SA approaches, respectively. Under the SA approach, we observe similar or increased experimentation at combinations with DLT probability within 5*%* of the TTL *Γ* = 0.25; scenario 6 shows that under the SA approach, over 16.5*%* of patients are dosed within this interval, relative to the 14.4*%* under the NA approach. When considering a wider probability interval of (0.2,0.3], one observes similar results, with fewer patients receiving doses with probability of DLT greater than 30*%* and 40*%*; again for scenario 6, 50.8*%* of patients receive combinations with DLT probabilities between 0.20 and 0.30, which is several percentage point below the SA approaches (53–54.3%). For scenarios 4 and 5, under the NA approach, we have 41.5*%* and 56.9*%*, respectively, which is less than nearly all SA approaches in the same scenarios (43.5–45.1% and 54.6–58.6%, respectively). Overall, the mean DLT rate for the SA approach (for all underlying values of *λ*_*TR*_ studied) is less than or equal to that under the NA approach, although Figure 4 illustrates that the mean DLT rate of each approach as the number of patients increases is fairly similar. Table 5 also shows the mean percentage (and standard deviation) of DLTs in each trial that occur before time *t*_*B*_. Although changes are small, it is shown that a slightly reduced DLT rate is observed under the SA approach when $\lambda_{\mathrm{TR}}=\frac{13}{14}$ relative to the NA approach when the percentage of DLT rates before time *t*_*B*_ is higher, which can be seen in all scenarios.

**Table 5:** Percentage of experimentation at combinations with DLT probabilities within different probability intervals and DLT rate (mean % and SD) for NA and SA approaches, and percentage of DLTs before time *t*_*B*_(mean % and SD) under SA approach, for various values of *λ*_*TR*_ under scenarios 1–6.

	Probability of DLT							DLT rate (%)		DLTs pre‐*t*_*B*_ (%)	
	[0,0.2]	(0.2,0.225]	(0.225,0.275]	(0.275,0.3]	(0.3,0.4]	(0.4,1]		Mean	SD	Mean	SD
Scenario 1											
NA	13.5	19.2	26.1	8.7	29.6	2.9		29.1	9.0	—‐	—
SA $\left(\lambda_{\mathrm{TR}}=\frac{2}{14}\right)$	12.0	16.9	26.5	13.5	28.4	2.8		29.4	9.0	2.2	2.6
SA $\left(\lambda_{\mathrm{TR}}=\frac{8}{14}\right)$	12.5	19.1	26.1	10.5	28.7	3.0		28.8	8.6	9.9	6.7
SA $\left(\lambda_{\mathrm{TR}}=\frac{13}{14}\right)$	12.5	18.6	27.5	10.7	27.7	3.0		28.8	8.8	16.1	8.2
Scenario 2											
NA	36.7	20.7	42.5	—	—	—		20.5	4.7	—	—
SA $\left(\lambda_{\mathrm{TR}}=\frac{2}{14}\right)$	38.3	20.8	40.9	—	—	—		20.3	4.6	1.4	1.5
SA $\left(\lambda_{\mathrm{TR}}=\frac{8}{14}\right)$	36.7	19.9	43.3	—	—	—		20.4	4.6	6.4	3.2
SA $\left(\lambda_{\mathrm{TR}}=\frac{13}{14}\right)$	37.0	20.3	42.7	—	—	—		20.3	4.6	10.7	4.7
Scenario 3											
NA	30.4	20.9	21.6	27.1	—	—		22.7	5.8	—	—
SA $\left(\lambda_{\mathrm{TR}}=\frac{2}{14}\right)$	31.3	20.3	22.0	26.3	—	—		22.5	5.6	1.9	1.7
SA $\left(\lambda_{\mathrm{TR}}=\frac{8}{14}\right)$	31.4	19.3	21.3	28.1	—	—		22.6	5.3	8.9	5.7
SA $\left(\lambda_{\mathrm{TR}}=\frac{13}{14}\right)$	32.5	19.9	20.9	26.7	—	—		22.5	5.4	13.9	6.2
Scenario 4											
NA	25.0	0.0	28.9	12.6	29.2	4.4		29.9	9.9	—	—
SA $\left(\lambda_{\mathrm{TR}}=\frac{2}{14}\right)$	21.1	0.0	29.0	14.8	30.9	4.2		30.1	9.7	3.2	3.3
SA $\left(\lambda_{\mathrm{TR}}=\frac{8}{14}\right)$	24.3	0.0	30.7	12.8	28.0	4.3		29.3	9.4	14.5	8.6
SA $\left(\lambda_{\mathrm{TR}}=\frac{13}{14}\right)$	23.8	0.0	32.6	12.5	27.5	3.7		29.7	10.0	24.4	10.8
Scenario 5											
NA	—	26.7	13.7	16.5	36.3	6.9		34.7	11.1	—	—
SA $\left(\lambda_{\mathrm{TR}}=\frac{2}{14}\right)$	—	26.4	8.0	20.2	39.1	6.4		34.6	10.7	4.1	5.1
SA $\left(\lambda_{\mathrm{TR}}=\frac{8}{14}\right)$	—	25.9	14.0	18.4	35.8	6.0		33.8	10.8	16.6	10.5
SA $\left(\lambda_{\mathrm{TR}}=\frac{13}{14}\right)$	—	26.0	14.7	17.9	35.7	5.7		34.6	11.1	28.6	12.1
Scenario 6											
NA	—	30.9	14.4	5.5	37.0	12.3		34.9	11.0	—	—
SA $\left(\lambda_{\mathrm{TR}}=\frac{2}{14}\right)$	—	26.2	16.7	12.4	33.5	11.1		34.8	11.1	1.3	2.9
SA $\left(\lambda_{\mathrm{TR}}=\frac{8}{14}\right)$	—	25.8	16.8	11.4	34.9	11.2		34.8	10.9	3.8	4.4
SA $\left(\lambda_{\mathrm{TR}}=\frac{13}{14}\right)$	—	26.6	16.5	9.9	35.6	11.3		34.6	10.9	6.9	5.9

DLT, dose‐limiting toxicity; NA, non‐attributable; SA, semi‐attributable.

Table 6 shows the percentage of trials recommending each dose combination after all patients have been evaluated for NA and SA method, respectively, as well as the mean bias and RMSE for model parameters *α*, *β* and *γ*. With respect to MTD recommendations and their true DLT probabilities, the SA approach when *λ*_*TR*_ equals $\frac{8}{14}$ or $\frac{13}{14}$ has in general higher recommendation percentages within (0.225,0.275] and the larger interval (0.2,0.3], and fewer recommendations at combinations with DLT probability greater than 30*%*, which can be seen in scenarios 4, 5 and 6. Under scenario 2, fewer trials stopped under the SA approach for all values of *λ*_*TR*_, and the SA approaches had more MTD combinations recommended in the interval (0.225,0.275] (25.5–27.1%) compared with the NA approach (23.5*%*).

**Table 6:** Percentage of MTD recommendations within DLT probability intervals, bias and RMSE around parameter estimates, and number of trials stopping early, not recommending an MTD (not including early stopping) and mean number of MTD recommendations for scenarios 1–6.

																Early	No	Mean
	Probability of DLT							Bias				RMSE				stop	MTD	MTDs
	(0,0.2]	(0.2,0.225]	(0.225,0.275]	(0.275,0.3]	(0.3,0.4]	(0.4,1]		*α*	*β*	*γ*		*α*	*β*	*γ*		(*N*)	(*N*)	(*N*)
[[LWMulCol]]1[[LWMulCol]] Scenario 1																
NA	1.9	16.7	34.7	17.8	28.5	0.4		0.14	0.05	0.02		0.23	0.20	0.21		132	8	2.2
SA $\left(\lambda_{\mathrm{TR}}=\frac{2}{14}\right)$	3.0	20.7	30.5	16.5	28.9	0.4		0.42	−0.15	0.14		0.47	0.23	0.23		132	28	1.7
SA $\left(\lambda_{\mathrm{TR}}=\frac{8}{14}\right)$	1.9	16.5	33.7	16.2	31.4	0.4		0.09	0.09	0.08		0.22	0.22	0.21		111	11	2.2
SA $\left(\lambda_{\mathrm{TR}}=\frac{13}{14}\right)$	2.2	15.9	33.5	14.1	33.9	0.4		−0.05	0.28	−0.09		0.16	0.35	0.24		120	6	2.3
Scenario 2																		
NA	38.8	37.7	23.5	—	—	—		0.06	0.05	1.81		0.17	0.18	1.83		8	288	1.4
SA $\left(\lambda_{\mathrm{TR}}=\frac{2}{14}\right)$	33.2	39.7	27.1	—	—	—		0.21	−0.07	2.03		0.23	0.21	2.04		7	244	1.3
SA $\left(\lambda_{\mathrm{TR}}=\frac{8}{14}\right)$	35.9	37.9	26.2	—	—	—		0.06	0.05	1.92		0.19	0.18	1.94		7	271	1.4
SA $\left(\lambda_{\mathrm{TR}}=\frac{13}{14}\right)$	34.5	40.1	25.5	—	—	—		−0.05	0.17	1.73		0.20	0.23	1.76		7	242	1.5
Scenario 3																		
NA	21.6	34.6	31.5	12.3	—	—		0.17	−0.13	−0.90		0.24	0.22	0.93		26	103	2.0
SA $\left(\lambda_{\mathrm{TR}}=\frac{2}{14}\right)$	20.1	28.2	36.2	15.4	—	—		0.35	−0.27	−0.72		0.37	0.34	0.74		22	81	1.7
SA $\left(\lambda_{\mathrm{TR}}=\frac{8}{14}\right)$	22.5	32.9	31.0	13.6	—	—		0.13	−0.09	−0.85		0.23	0.20	0.88		18	93	2.1
SA $\left(\lambda_{\mathrm{TR}}=\frac{13}{14}\right)$	20.7	34.1	33.0	12.1	—	—		0.00	0.06	−1.06		0.19	0.17	1.10		20	93	2.2
Scenario 4																		
NA	10.2	0.0	35.7	16.7	36.7	0.7		0.30	−0.28	2.00		0.35	0.35	2.01		165	9	2.1
SA $\left(\lambda_{\mathrm{TR}}=\frac{2}{14}\right)$	14.5	0.0	31.0	16.3	37.3	0.8		0.52	−0.48	2.15		0.56	0.53	2.15		163	35	1.7
SA $\left(\lambda_{\mathrm{TR}}=\frac{8}{14}\right)$	10.3	0.0	39.2	19.9	29.9	0.7		0.16	−0.11	1.94		0.23	0.23	1.95		147	13	2.1
SA $\left(\lambda_{\mathrm{TR}}=\frac{13}{14}\right)$	11.1	0.0	40.1	19.8	28.3	0.6		0.04	0.17	1.63		0.14	0.22	1.66		184	19	1.9
Scenario 5																		
NA	—	12.4	14.1	28.9	41.6	3.0		0.29	−0.29	0.02		0.35	0.36	0.24		345	23	1.3
SA $\left(\lambda_{\mathrm{TR}}=\frac{2}{14}\right)$	—	15.7	12.9	24.0	43.5	3.9		0.50	−0.48	0.14		0.55	0.51	0.23		339	37	1.1
SA $\left(\lambda_{\mathrm{TR}}=\frac{8}{14}\right)$	—	10.3	16.9	30.3	40.6	2.0		0.17	−0.13	−0.02		0.22	0.24	0.22		320	23	1.4
SA $\left(\lambda_{\mathrm{TR}}=\frac{13}{14}\right)$	—	15.0	16.5	28.4	38.7	1.4		0.05	0.20	−0.27		0.12	0.25	0.39		383	27	1.2
Scenario 6																		
NA	—	14.8	21.1	11.1	48.7	4.3		−0.34	0.27	−0.97		0.39	0.31	1.00		363	29	1.1
SA $\left(\lambda_{\mathrm{TR}}=\frac{2}{14}\right)$	—	16.7	22.9	13.1	44.3	3.0		0.04	0.09	−0.86		0.18	0.14	0.88		371	55	0.9
SA $\left(\lambda_{\mathrm{TR}}=\frac{8}{14}\right)$	—	14.3	20.4	15.2	46.9	3.2		−0.09	0.14	−0.87		0.23	0.19	0.89		358	37	1.0
SA $\left(\lambda_{\mathrm{TR}}=\frac{13}{14}\right)$	—	12.1	21.1	14.4	48.1	4.3		−0.23	0.21	−0.91		0.32	0.26	0.93		341	38	1.1

DLT, dose‐limiting toxicity; NA, non‐attributable; SA, semi‐attributable; RMSE, root mean‐squared error.

With respect to the bias and RMSE, it is observed that the mean bias and RMSE for the interaction parameter *γ* are fairly similar across all methods per scenario and the bias seems to indicate that the final parameter estimates are close to 0; given that the prior on *γ* is reasonably vague, it is likely that changes in the dose‐toxicity surface are determined by the marginal parameters *α* and *β*. The results for RMSE on scenarios 4, 5 and 6 suggest that when toxicity increases slowly for one drug at the marginal level (seen in drug *B* for scenarios 4 and 5, and drug *A* for scenario 6), more precise parameter estimates are obtained, because more experimentation is permitted at increasing dose levels of that drug. The converse can be seen for the RMSE around the parameter relating to the other agent, which in truth has DLT probability rate increasing much faster.

### 5.3 Sensitivity analysis

We also conducted a sensitivity analysis to assess how the SA approach performs relative to the NA approach when *λ*_*TR*_ is increasing with *a*_*j*_, that is, higher dose levels of drug *A* have an increased probability of DLT in time window [0,*t*_*B*_) relative to the case where *λ*_*TR*_ is constant for all *a*_*j*_. We compared both approaches on scenario 5, so the true probabilities of DLT over the interval [0,*T*] were identical to those given for scenario 5 in Table 2, but with *λ*_*TR*_(*a*_*j*_) = 1/14 for *j* = 1, 3/14 for *j* = 2, 5/14 for *j* = 3 and 8/14 for *j* = 4. Therefore, the probability of DLT due to drug *A* was not directly proportional to the marginal probabilities of DLT at each dose of drug *A*, as previously assumed. This gave the probabilities of DLT in time window [0,*t*_*B*_) at (*a*_1_,*a*_2_,*a*_3_,*a*_4_) as (0.01, 0.06, 0.12 and 0.22). We simulated 1000 trials per approach, under the same conditions detailed in Subsection 4.4 (results are provided in Supporting Information to this paper). We found a trade‐off between experimentation and MTD recommendation, with the SA approach identifying the MTD combination (in this scenario, (*a*_1_,*b*_2_)) 1.9% more than the NA approach; furthermore, the NA approach recommended 1.5% more overdoses (DLT probability over [0,*T*] greater than 0.30) than the SA approach. However, with regard to experimentation, 1.7% more patients received the true MTD combination under the NA approach than the SA approach, and 1% more patients received combinations with true DLT probability greater than 0.40 under the SA approach.

## 6 Conclusions

In this paper, modifications to standard dual‐agent dose‐escalation methodology that may be used for clinical trials with non‐concurrent administration of agents over a cycle have been investigated. By changing the structure of the likelihood and modelling the response as a trinary categorical variable, rather than a simple binary variable, improvements to the performance of model‐based trial design are observed in several scenarios, including slight increases in the percentage of patients receiving dose combinations with true DLT probabilities close to the TTL *Γ*, reductions in the percentage of patients receiving dose combinations with true DLT probabilities much higher than the TTL *Γ* and the distribution of MTD recommendations at the end of the trial. For the simulation study in Sections 4 and 5, the SA approach dosed more patients at target combinations and fewer patients at overdoses relative to the NA method. However, the SA approach did not universally outperform the NA method. Also, the SA approach recommends target combinations (or those close to target combinations on the probability scale) more often than the NA method.

However, we acknowledge that there are limitations with the work presented here. As stated previously, the submitted protocol includes a 2000mg/100ml fixed dose of gemcitabine to be administered on day 3, between the administration of Cabazitaxel (day 1) and Cisplatin (day 5). The methodology introduced here is intended to incorporate dual‐agent dose‐escalation methodology into statistical model‐based trial design, and therefore focus on the two agents with adjustable dose levels as per the submitted protocol. The addition of a third agent, albeit a fixed dose that has shown to be well‐tolerated in patients at the proposed concentration, introduces further complexity into the modelling framework; Yin and Yuan 21 acknowledge such an extension via the use of copula regression. Such an expansion to a three‐agent dose‐escalation problem, along with exploration of new methodology for SA toxicity, is particularly challenging and requires an extremely detailed analysis of operating characteristics. Furthermore, we only consider drug‐related toxicities in our methodology and not disease‐related toxicity, or the problem of toxicity misattribution 11. However, as mentioned in Section 1, both drugs will have been studied separately in single‐agent phase I trials, so misattributing disease‐related toxicity to drugs and vice versa is less likely than in monotherapy trials.

A further consideration is the choice of model for modelling the dose‐toxicity surface. As stated previously, the choice of model used in this research was made because of its parsimony and also its ability to satisfy all of the aforementioned assumptions in Section 3, as well as its ability to model different forms of interactive behaviour. It does not serve as a formal recommendation for this particular model, and other binary regression models are available for use in dual‐agent phase I dose‐escalation trials 12. Before deciding how to model the dose‐toxicity surface, investigators and statisticians should discuss the various aspects of a proposed trial in order to consider all possible options for the trial conduct. It may be the case that other dose‐toxicity models proposed in the literature, novel extensions of these or indeed entirely new methodology developed specifically for a particular trial will serve as the best method 17. Further, to the research shown here, we also considered setting $P\left(Y_{i}=2|a_{j},b_{k},t_{B},\psi \right)=(1-\lambda )\pi_{T}\left(a_{j},b_{k},\theta \right)$ and expressing the probability of DLT as $P\left(Y_{i}=1|a_{j},t_{B},\psi \right)+P\left(Y_{i}=2|a_{j},b_{k},t_{B},\psi \right)$, then assessing how end‐of‐trial MTD recommendations differed; there was very little difference between those shown under the proposed model in equations (3), (4) and (5).

One key point of discussion is the assumption that $\pi_{t_{B}}\left(a_{j};\psi \right)=\lambda \pi_{T}\left(a_{j},0;\theta \right)$. This assumes that the probability of DLT in the time interval [0,*t*_*B*_) is linearly proportional to the probability of DLT in the time interval [0,*T*] at dose combination (*a*_*j*_,0). This is a rather neat and simple assumption regarding the nature of the dose‐toxicity relationship between the two drugs. The sensitivity analysis in Subsection 5.3 investigated model performance when this assumption was not true for one scenario and found that the SA approach was better than the NA approach at correctly identifying MTD combinations and selecting doses near the MTD, but the NA approach was slightly better with regard to experimentation. Perhaps a more advanced idea would be that the time to a DLT occurring is based on some exponential distribution, dependent on the type and number of drugs given. One may instead assume $\pi_{t_{B}}\propto e(\pi_{T})$ that is $\pi_{t_{B}}$ is proportional to some other function *e* of *π*_*T*_ that may not be linear. Alternatively, $\pi_{t_{B}}$ may not be related to *π*_*T*_ at all, requiring two different probability functions to be chosen for $\pi_{t_{B}}$ and *π*_*T*_. However, because dose‐escalation decisions are made based on function *π*_*T*_ alone, this would require some modification to the function that determines which dose combination to give to the next cohort, and indeed to recommend at the end of the trial. As it stands, the current simplifying assumption of $\pi_{t_{B}}\left(a_{j};\psi \right)=\lambda \pi_{T}\left(a_{j},0;\theta \right)$ seems sensible and reduces the complexity of this dose‐escalation approach. With respect to accuracy of dose‐toxicity modelling, it may be the case that the choice of probability function *π*_*T*_ has a far bigger role to play in dose‐toxicity modelling than the linking postulation of the relationship between probability functions $\pi_{t_{B}}$ and *π*_*T*_.

Based on the work conducted in this paper, the incorporation of methodology relating to SA toxicity may be applied to dual‐agent trials that incorporate non‐concurrent drug administration but tailored to the specific trial. If information relating to non‐overlapping toxicities is known, or if the clinician can distinguish drug‐related toxicity from disease‐related toxicity, then the dose‐toxicity model may be modified so that such information can be used to guide dose‐escalation/de‐escalation. Furthermore, if pharamcokinetic/pharmacodynamic data can be used to help predict the probability of DLT occurring over a particular interval, and inform the potential for carry‐over effects both at the point of administering drug *B* and even between cycles, then this could be incorporated to make dose‐escalation methods more advanced and realistic. The work presented here marks a novel and firm starting point for considering individual trial aspects to tailor advanced Bayesian methodology to a clinical research question of interest.

Considering the results obtained and the limitations identified, further areas of research can be explored. Aside from modifications already addressed such as model choice, assumptions linking $\pi_{t_{B}}$ and *π*_*T*_ and the inclusion of more than two drugs in the model, it would be particularly interesting to consider how multiple toxicities and their gradings influence dose‐escalation/de‐escalation decisions for drugs administered non‐concurrently. An additional point of interest would be to consider the occurrence of DLTs outside of the first cycle of treatment, where DLT responses are traditionally recorded, particularly in a dual‐agent trial with non‐concurrent drug administration. A tougher practical consideration would be to see if the time of administration of drug *B* could be adapted during the trial so that more patients are given the full combination, though still keep the sequential administration structure.

## Supporting information

Supporting Information
