# Supplementary material for: Modelling semi‐attributable toxicity in dual‐agent phase I trials with non‐concurrent drug administration
Source: Stat Med. 2016 Feb 19;36(2):225–41. doi: 10.1002/sim.6912 (PMC5157785; doi:10.1002/sim.6912)
Supplement: Supplementary file 1 — Supporting Information [file SIM-36-225-s001.pdf]

	Probability of DLT						DLT Rate (%)		DLTs pre-t_B (%)	
	[0,0.2]	(0.2,0.225]	(0.225,0.275]	(0.275,0.3]	(0.3,0.4]	(0.4,1]	Mean	SD	Mean	SD
NA	-	38.8	16.3	17.9	25.1	1.8	12.2	6.5	-	-
SA	-	39.7	14.6	19.6	23.2	2.8	12.9	5.7	4	4.2

Table S1: Percentage of experimentation at combinations with DLT probabilities within different probability intervals and DLT rate (mean % and SD) for Non-Attributable (NA) and Semi-Attributable (SA) approach, and percentage of DLTs before time t_B (mean % and SD) under SA approach, for $\boldsymbol{\lambda}_{TR} = (1/14, 3/14, 5/14, 8/14)$.

	Probability of DLT						Bias			RMSE			Early Stop	No MTD	Mean MTDs
	(0,0.2]	(0.2,0.225]	(0.225,0.275]	(0.275,0.3]	(0.3,0.4]	(0.4,1]	α	β	γ	α	β	γ	(N)	(N)	(N)
NA	-	22.7	21.9	33.2	21.9	0.3	0.19	-0.31	0.05	0.24	0.37	0.23	435	37	0.9
SA	-	23.3	23.8	32.3	20.4	0.3	0.38	-0.68	0.11	0.42	0.70	0.19	426	29	1.1

Table S2: Percentage of MTD recommendations within DLT probability intervals, bias and root mean squared error (RMSE) around parameter estimates, and number of trials stopping early, not recommending an MTD (not including early stopping) and mean number of MTD recommendations, for $\boldsymbol{\lambda}_{TR} = (1/14, 3/14, 5/14, 8/14)$.
